# COVID-19 risk perception and public compliance with preventive measures: Evidence from a multi-wave household survey in the MENA region

**DOI:** 10.1371/journal.pone.0283412

**Published:** 2023-07-10

**Authors:** Assem Abu Hatab, Lena Krautscheid, Franklin Amuakwa-Mensah

**Affiliations:** 1 Nordic Africa Institute, Uppsala, Sweden; 2 Department of Economics, Swedish University of Agricultural Sciences, Uppsala, Sweden; 3 Department of Design Sciences, Faculty of Engineering LTH, Lund University, Lund, Sweden; 4 Environment for Development, University of Gothenburg, Gothenburg, Sweden; 5 Department of Social Sciences, Technology and Arts, Luleå University of Technology, Luleå, Sweden; UV: Universiteti Ismail Qemali Vlore, ALBANIA

## Abstract

This study investigates the association between individuals’ concern about contracting COVID-19 and their compliance with recommended preventive and mitigation measures, namely wearing face masks, maintaining social distancing and handwashing, in the context of the Middle East and North Africa (MENA) region. The empirical analysis is based on a panel dataset from the Combined COVID-19 MENA Monitor Household Survey, which was carried out in Jordan, Morocco, Sudan, Tunisia and Egypt. Applying a probit estimation technique, a positive and statistically significant association was found between the level of COVID-19 worries and individuals’ compliance with the mitigation measures. Notably, the results revealed that this association followed a “first-up-then-down” trend, showing that compliance with the three mitigation measures rose as individuals’ worries about contracting the virus increased, and then markedly decreased after they had been infected. Socio-demographic characteristics contributing to lower levels of compliance included being male, being over 60, having lower levels of education and having a lower household income. A cross-country analysis revealed remarkable differences between the five countries, with the strongest association between COVID-19 concerns and adherence to mitigation measures observed in Tunisia and Sudan, and the weakest association seen in Jordan and Morocco. Policy implications are outlined for effective risk communication and management during disease outbreaks and public health emergencies to encourage appropriate public health behaviours.

## 1. Introduction

As elsewhere around the globe, the spread of the coronavirus disease 2019 (COVID-19) posed formidable challenges to healthcare systems in the Middle East and North Africa (MENA) region. With the first case confirmed in February 2020, the virus rapidly spread through countries in the region (that is, Algeria, Bahrain, Egypt, Iran, Iraq, Israel, Jordan, Kuwait, Lebanon, Libya, Morocco, Oman, Palestine, Qatar Saudi Arabia, Syria, Tunisia, United Arab Emirates and Yemen), causing more than 224,000 confirmed cases and nearly 8,400 deaths by May 2020 [1]. As at February 2023, the WHO Coronavirus Dashboard shows that MENA countries have reported more than 26 million confirmed cases and over 323,000 deaths [1]. Generally, the severe spread of the virus in MENA countries has been attributed to the influence of a suite of institutional and contextual factors, including the healthcare systems’ lack of preparedness, the inadequacy of health resources and management strategies, governments’ denial of the seriousness of infection among the population in the early stages of the pandemic, and widespread conflict and migration in the region that hampered individuals’ access to healthcare [2, 3]. In addition, evidence has accumulated to show that the public’s non-compliance with MENA governments’ preventive measures (e.g. social distancing, mandatory use of face masks, and repeated handwashing) contributed to the spread of the virus in MENA countries, as well as to the emergence of severer subsequent waves of infection in late 2020 and 2021. This turned many countries into epicentres for viral spread, leading to high mortality rates and damaging social and economic repercussions [4–7].

From a health-behaviour theory perspective, risk perception, defined as an individual’s perceived susceptibility to a threat, is a key factor in shaping individuals’ health behaviour and a predictor of such behaviours being maintained [8, 9]. In other words, the extent to which individuals believe that COVID-19 infection is severe and likely to affect their health should predict their intention and actual behaviours in relation to compliance with risk prevention and mitigation measures, such as using a face mask, maintaining social distancing and frequent handwashing [9]. Therefore, developing a deeper understanding of how the perceived severity of and susceptibility to threats influence people’s behavioural responses and compliance with governments’ mitigation measures during times of crises (*e*.*g*. the COVID-19 pandemic) is crucial in order to formulate effective public health strategies for risk prevention and mitigation [10].

Building on the Health Belief Model (HBM) developed by Rosenstock [11], and validated in a large body of empirical studies [12], the present study investigated the association between individuals’ concern about contracting COVID-19 and their engagement in three preventive behaviours, namely wearing face masks, maintaining social distancing and handwashing. The study used panel dataset from five MENA countries (Egypt, Jordan, Morocco, Sudan, and Tunisia) with 34,219 observations, and employed a probit estimation technique to empirically examine this association, while controlling for country and survey wave fixed effects, socio-demographic characteristics (gender, age, income and education level) and community characteristics (rural *versus* urban areas).

Specifically, the study addressed three important limitations in existing literature on the association between risk perception as behavioural triggers and preventive behaviours in the context of the COVID-19 pandemic. First, despite the extensive literature published on risk perception [13, 14] and preventive behaviours [15, 16] individually, very few studies consider the association between them. In connection with this, the level of an individual’s concern about infection has received very little attention in this literature [17] compared with other behavioural triggers such as knowledge and attitudes [18] or psychological disorders such as anxiety and depression [19], despite the crucial role played by individuals’ level of concern about infection in shaping their health behaviours [20]. Thus there is a research gap with regard to understanding the influence of people’s level of perception on their subsequent preventive behaviours.

Second, most of the earlier studies have focused on a single wave of the pandemic, neglecting the evidence showing that individuals’ perception and concern about COVID-19 infection, and thus their protective behaviour, underwent substantial changes over the course of the pandemic and across various waves [21]. Such changes largely depended on infection rates, media coverage and public policy and communication in relation to the implementation of migration measures [22]. Our study used multiple waves of the Combined COVID-19 MENA Monitor Household survey spanning from November 2020 to August 2021, and controlled for country-specific and time trends. This enabled changes within a country over time that may be associated with the implementation of risk preventive measures and changes in perceptions and concern among the population to be accounted for.

The third limitation is related to the overrepresentation of online surveys together with small sample sizes, which can be attributed to the conditions of lockdown and restricted travel that largely constrained the collection of primary data. The present study “partially” addressed this limitation by using a panel dataset collected by means of telephone surveys with 34,219 individuals from five MENA countries, which provided comprehensive coverage and a significantly larger sample size than have been found in existing studies [23, 24]. Potential respondents with mild or no interest can ignore or delete e-mailed or online requests to complete an online survey, but a trained telephone interviewer can encourage those who are not enthusiastic about completing the survey to participate [25]. Therefore, it is anticipated that the use of a telephone survey together with the large sample size on which the empirical analysis in this paper is based will mitigate concerns associated with the sample representativeness of web-based and online surveys.

The section below sets out the methodology and data used in the empirical analysis. Section 3 reports and discusses the empirical findings, while Section 4 summarises the study and concludes by considering implications for further research.

## 2. Materials and methods

### 2.1. Theoretical background and empirical model

The theoretical underpinnings of this study lean on the health belief model (HBM), which provides a widely accepted framework for understanding and analysing health behaviours [26, 27]. The general HBM assumption is twofold: *i*) health-related behaviours are closely associated with the level of concern and anxiety perceived by an individual as a risk or threat, and *ii*) it is anticipated that the benefits that an individual would gain from performing such proactive behaviours manage the given threat effectively and outweigh any obstacles to implementing them [28]. Within HBM analytical frameworks, risk prevention or reduction behaviours represent the dependent construct, which is hypothesised to be influenced by individuals’ perceptions (e.g. level of concern and perceived severity and susceptibility), and a set of modifying factors comprising demographic characteristics and health-related habits and knowledge.

In line with HBM assumptions and considering the nature of the variables included in the panel dataset, concern about COVID-19 infection was used as a proxy for the perceived threat of the pandemic. Compliance with preventive measures by undertaking preventive behaviours, such as washing hands, avoiding mass gatherings and wearing face masks, depended on the level of risk that individuals perceived during different waves of the pandemic. The modifying factors consisted of the following demographic characteristics: gender, age, income, education level and community characteristics captured by the rurality or urbanity of the area in which the individual resides. Thus, the drivers of COVID-19 preventive measures (i.e. the wearing of face masks, social distancing and handwashing) were modelled as a function of infection worries, socio-economic and demographic factors, time invariance factors, and country and administrative-level fixed effects, following Amuakwa-Mensah et al. [29]. The empirical model that explored the relationship between compliance with COVID-19 preventive measures and infection worries is shown in Eq (1):

$${y*}_{ijkt}=\beta_{0}+\beta_{1}{Covid\_worries}_{ijkt}+X_{ijkt}\beta +{\mu_{jk}+\eta }_{k}+\gamma_{t}+\varepsilon_{ijkt}$$ (1)

where *y*_*ijkt*_ is the probability of an individual (*i*) in community (*j*) residing in country (*k*) adhering to COVID-19 preventive measures at time (*t*). The outcome variables of interest, i.e. the wearing of a face mask, social distancing and handwashing, are binary. It takes the value of 1 if an individual adheres to a specific COVID-19 preventive measure at a point in time, and 0 otherwise. In the case of mask wearing, respondents were asked to indicate whether or not they wear a mask when they are outside the house. For social distancing, respondents were asked to indicate whether they stay at least one metre away from people when they are outside their house. Similarly, respondents were asked to indicate if they wash their hands with soap more often than they did before COVID-19. The term *Covid*_*worries* represents the level of concern an individual has about contracting COVID-19. Individuals were asked to indicate how worried they were about being infected with COVID-19. Respondents could choose between the following options: “not worried”, “a little worried”, “rather worried”, “very worried” and “already infected”. The term ***X***_*ijkt*_ is a vector representing socioeconomic and demographic factors, and other control variables such as household size, age, education, marital status, employment status, income quartile and locality (i.e. urban or rural). In addition, survey wave (*γ*_*t*_), country (*η*_*k*_) and administrative (*μ*_*jk*_) fixed effects were controlled for in the model. The disturbance term is captured as *ε*_*ijkt*_.

Given the binary nature of the outcome variables, Eq (1) was estimated based on a probit model using the maximum likelihood estimation technique. From Eq (1), while y*_ijkt_ is an unobservable (or latent) outcome, *y*_*ijkt*_ was observed, such that:

$$y_{ijkt}=\left\{ \begin{array}{l} 1\;if\;{y*}_{ijkt}>0\\ 0\;if\;{y*}_{ijkt}\leq 0 \end{array}\right.$$ (2)

where the zero threshold is a normalisation that is independent of whether the model in Eq (1) includes an intercept or not. Given the latent variable models in Eqs (1) and (2), the model could be reformulated as:

$$p=Pr(y_{ijkt}=1)=Pr(z^{\prime }\beta +\varepsilon_{ijkt}>0)=Pr(-\varepsilon_{ijkt}<z^{\prime }\beta )=F(z^{\prime }\beta )$$ (3)

where is *p* the probability of an event happening, the function *F*() is the cumulation density function (cdf) of −*ε*_*ijkt*_, and the term ***z*** is a vector representing the explanatory variables (i.e. terms ***X***_*ijkt*_ and *Covid*_*worries*), as discussed earlier. The vector ***β*** represents the respective coefficient of the explanatory variables in the model. Assuming the error term (*ε*_*ijkt*_) has a standard normal distribution, Eq (3) yields a probit model that is estimated using the maximum likelihood estimation technique.

For ease of interpreting the estimated coefficients from this model, the marginal effects after the maximum likelihood estimation were calculated. Formally, the marginal effect of a specific explanatory variable (τ) is estimated as follows:

$$\frac{\partial p}{\partial z_{\tau }}=\phi (z^{\prime }\beta )\beta_{\tau }$$

where *ϕ*() represents the standard normal density function.

Based on Eq (1), the coefficient of interest is *β*_1_, which measures the association between compliance with Covid-19 mitigation measures and infection worries. Given the categorical nature of *Covid*_*worries*, the option “not worried” was used as the reference category in the estimation. The possible endogeneity of the variable of interest (i.e. *Covid*_*worries*) is acknowledged, thus care should be taken when claiming causality in interpreting the results. Potential endogeneity could not be addressed due to data limitation in finding a valid instrumental variable. In addition to the full-sample analysis, sub-sample analysis was also considered focusing on gender, age group, education level, income quartiles, urban-rural and country-specific analysis to account for potential heterogeneity (variation) in relation to the association between adherence to COVID-19 preventive measures and infection worries.

### 2.2. Data and sampling design

This study was based on data from five waves of a Combined COVID-19 MENA Monitor Household survey (CCMMHH) carried out by the Economic Research Forum (ERF) between November 2020 and August 2021 [30]. The CCMMHH survey comprises a suite of panel telephone surveys that are rolled out approximately every two months. Experienced survey research and polling companies in each country conducted the interviews using computer-assisted telephone interviewing techniques. Specifically, the data consist of the base wave of the survey (November 2020) and four panel datasets collected in February 2021, April 2021, July 2021 and August 2021. Interviewers sought the consent of respondents on the telephone to their participation in the survey during each survey wave. After introducing the study to the respondents, they were informed that participation was voluntary and asked if they agreed to participate in this survey. The CCMMHH questionnaires covered a range of topics including demographic and household characteristics, labour market and employment characteristics, and income sources and levels. Furthermore, the questionnaires contained a module on attitudes to Covid-19 risks and adherence to the mitigation measures that were implemented during the pandemic in the surveyed MENA countries to contain the spread of the virus, which comprised wearing a face mask, maintaining social distancing and washing hands. Specific questions on all the modules of the questionnaire are provided by the Economic Research Forum, which is responsible for the Combined COVID-19 MENA Monitor Household survey (CCMMHH) [30].

The MENA countries covered by the survey are Egypt, Jordan, Morocco, Tunisia and Sudan. These countries did not appear in all the waves; thus, each country has a different baseline and follow-up periods. For each country and wave, the corresponding sample is shown in S1 Table. A stratified sampling design was used based on mobile operators’ country-specific market shares. The sample was designed to cover at least 2,000 unique households and individuals, focusing on mobile phone users aged 18–64 and using random digit dialling within the range of valid numbers. The selected number is dialled up to three times and unanswered phone numbers or those who answered but could not complete the survey were dropped from the study. More information on the sampling design and response and attrition rates is provided by the Economic Research Forum, which is responsible for the CCMMHH survey [30].

Respondents from previous waves were contacted again for a follow-up interview only if they had agreed to this in the earlier wave. Similarly, up to three call attempts were made to respondents for the follow-up interview and, in some cases, second and family/friends’ numbers were also contacted in an attempt to reach the respondent. In the event that an individual refused to respond or was unreachable for the follow-up interview, the individual was replaced by a new person who was randomly selected in accordance with the base wave protocols. In order to reduce sampling bias, an inverse probability weighting was applied based on the following: a) telephone operators and their market shares, b) number of telephones by operator for individuals and household members, and c) representative in-person survey data with comparable demographic and household characteristics. More details can be found on the ERF website in this link: http://www.erfdataportal.com/index.php/catalog/230/download/3459.

The descriptive statistics of the variables used in this study are shown in Table 1. Generally, a greater proportion (around 80%) of individuals in the MENA region adhered to COVID-19 containment measures such as social distancing, mask wearing and handwashing. However, Sudan had a relatively low percentage of individuals adhering to social distancing (56%) and wearing a mask (62%). The average age of the sample was around 36 years, and the household size averaged five individuals. The sample comprised about 41% females, and most of the sample lived in urban centres (73%). Egypt had almost an equal proportion of urban and rural dwellers. In relation to COVID-19 concerns, a higher percentage of individuals indicated that they were not worried about being infected with COVID-19, with Sudan having the highest number of individuals. On average, about 4% of respondents indicated that they had already been infected with COVID-19. However, 7.8% of respondents in Jordan had been infected by COVID-19 and only 1% in Sudan indicated that they had already been infected by the virus.

**Table 1:** Descriptive statistics of the CCMMHH survey sample.

VARIABLES	All		Jordan	Morocco	Sudan	Tunisia	Egypt
	*Mean*	*Std*.	*Mean*	*Mean*	*Mean*	*Mean*	*Mean*
**Social distance**	0.834	(0.372)	0.883	0.880	0.563	0.877	0.865
**Mask wearing**	0.865	(0.342)	0.936	0.927	0.624	0.858	0.885
**Handwashing**	0.847	(0.360)	0.812	0.915	0.765	0.849	0.863
**Household size**	5.124	(2.433)	5.381	5.010	6.265	4.521	4.743
**Age**	36.46	(12.14)	36.53	37.19	30.20	40.00	35.15
**Female**	0.414	(0.493)	0.473	0.359	0.480	0.403	0.365
**Urban**	0.723	(0.447)	0.812	0.714	0.831	0.691	0.518
**COVID-19 concern**							
*Not at all worried*	0.388	(0.487)	0.319	0.426	0.479	0.377	0.356
*A little worried*	0.216	(0.411)	0.188	0.264	0.145	0.267	0.152
*Rather worried*	0.151	(0.359)	0.212	0.0750	0.157	0.143	0.203
*Very worried*	0.207	(0.405)	0.202	0.212	0.209	0.177	0.256
*Already infected*	0.0385	(0.192)	0.0780	0.0224	0.0102	0.0358	0.0337
**Educational attainment**							
*Less than basic*	0.223	(0.416)	0.105	0.380	0.105	0.268	0.171
*Basic* *	0.184	(0.388)	0.281	0.183	0.104	0.169	0.127
*Secondary*	0.325	(0.468)	0.326	0.183	0.411	0.353	0.466
*Higher education*	0.267	(0.443)	0.288	0.254	0.380	0.211	0.236
**Marital status**							
*Never married*	0.319	(0.466)	0.248	0.326	0.538	0.295	0.240
*Currently married*	0.635	(0.481)	0.698	0.625	0.431	0.661	0.715
*Widowed/divorced*	0.0460	(0.209)	0.0537	0.0495	0.0316	0.0438	0.0444
**Employment status**							
*Employed*	0.511	(0.500)	0.439	0.508	0.401	0.616	0.581
*Unemployed*	0.234	(0.423)	0.272	0.193	0.306	0.213	0.207
*Out of the labour force*	0.255	(0.436)	0.289	0.299	0.294	0.171	0.212
**Income quartile**							
*First quartile*	0.253	(0.435)	0.257	0.363	0.0941	0.199	0.299
*Second quartile*	0.255	(0.436)	0.350	0.257	0.167	0.200	0.271
*Third quartile*	0.199	(0.399)	0.193	0.107	0.184	0.294	0.234
*Fourth quartile*	0.175	(0.380)	0.159	0.0470	0.395	0.233	0.113
**Don’t know**	0.106	(0.308)	0.0323	0.206	0.147	0.0690	0.0674
**Refused to answer**	0.0125	(0.111)	0.00816	0.0204	0.0134	0.00592	0.0157
**Observations**	31,436		7,471	8,120	4,401	7,437	4,007

Note: Std. represents standard deviation.

* In Egypt basic education comprises primary education covering grades 1–9. In Jordan it entails grades 1–10. In Morocco it comprises grades 1–9. In Sudan it comprises eight years of primary education years (i.e. grades 1–8). In Tunisia it entails pre-school and grades 1–9.

## 3. Empirical results

### 3.1. Full-sample estimates

Table 2 presents the marginal effect based on the three probit model estimations, which were estimated to examine the association between the level of individuals’ concern about contracting COVID-19 and their compliance with their government’s risk prevention and mitigation measures. A deeper look at the estimates related to the independent variable (concern about infection) in the estimated models for maintaining social distancing, using a face mask and frequent handwashing, revealed two interesting findings. First, the results demonstrated that the adoption of COVID-19 mitigation measures was positively associated with the levels of perceived concern about infection, while the coefficients of this variable in three estimated models were positive and highly statistically significant at the 1% level.

**Table 2:** Full-sample model estimates of determinants of compliance with COVID-19 mitigation measures.

Variables	Estimated models		
	Social distancing	Face mask	Handwashing
**Worried about infection (ref: Not worried)**			
*A little worried*	0.090***	0.081***	0.089***
	(0.004)	(0.004)	(0.004)
*Rather worried*	0.106***	0.089***	0.115***
	(0.004)	(0.004)	(0.004)
*Very worried*	0.120***	0.102***	0.129***
	(0.004)	(0.003)	(0.004)
*Already infected*	0.056***	0.042***	0.061***
	(0.008)	(0.007)	(0.007)
*Household size*	-0.003	-0.080***	-0.001
	(0.007)	(0.001)	(0.001)
**Age**	0.002***	0.002***	0.001***
	(0.000)	(0.000)	(0.000)
**Female**	0.040***	0.049***	0.043***
	(0.005)	(0.004)	(0.005)
**Urban**	-0.003	-0.000	-0.006
	(0.005)	(0.004)	(0.005)
**Education (ref: Less than basic)**			
*Basic*	-0.012*	-0.018***	-0.017**
	(0.007)	(0.006)	(0.007)
*Secondary*	-0.002	0.006	-0.003
	(0.006)	(0.005)	(0.006)
*Higher education*	0.004	0.012**	0.016***
	(0.007)	(0.006)	(0.007)
**Observations**	31,429	31,433	31,432
**Controls**	YES	YES	YES
**Country & Admin FE**	YES	YES	YES
**Wave FE**	YES	YES	YES
**Pseudo R** ^**2**^	0.146	0.180	0.0937
**Wald chi** ^**2**^	3640	3739	2284

Standard errors in parentheses

*** p<0.01

** p<0.05

* p<0.1. Marital status, employment status, income quartile, wave, country and administrative fixed effects were controlled for in all the models

Second, the marginal effect of adherence to COVID-19 mitigation measures in the three models was found to rise as the level of individuals’ concern grew from “a little worried” to “very worried”, and then markedly decreased when individuals were infected with the virus. For instance, in the social distancing model, the value of the concern coefficient rose from 0.09 for the variable category “a little worried” to 0.11 and 0.12 for the variable categories of “very worried” and “rather worried” respectively, and then nearly halved (0.06) for the “already infected” category. Similarly, the values of the coefficient rose from 0.08 and 0.09 for the “a little worried” category of the sample in the face-mask wearing and handwashing models to 0.09 and 0.11 respectively for the “rather worried” category. After reaching their maximum value for the “very worried” category (0.10 and 0.13), they then fell below the baseline level of the reference group (not worried) for the “already infected” category of the sample.

In addition, the parameters of the individual attributes (age, gender and education) and household size reported in Table 2 indicated that demographic and household characteristics were important determinants of compliance with public health measures. In particular, females were found to be significantly more likely to comply with the three mitigation measures than their male counterparts. Likewise, the results revealed that adoption of protective behaviours increased with age. In contrast, the association between formal educational attainment and compliance with COVID-19 mitigation measures was rather tenuous and to some extent paradoxical. For instance, individuals with a basic level of education were found to be significantly less likely to use face masks, practise repeated handwashing, and maintain social distancing during the COVID-19 pandemic than others with a less than basic level of education.

Nevertheless, the results highlighted that individuals with higher educational degrees were significantly more likely than those with a less than basic level of education to wear face masks and practise repeated handwashing during the COVID-19 pandemic. Despite the statistical insignificance of the coefficients related to social distancing and handwashing, in terms of household factors, the negative sign of the coefficient of household size in the three models suggested that a larger household size generally hampered the ability of individuals to comply with mitigation measures. Finally, no statistically significant evidence was found of the impact of the characteristics of the community (rural vs. urban areas) in which an individual resides on compliance with COVID-19 preventive and mitigation measures.

### 3.2. Heterogeneity analysis

Given the possible variation in the association between concern about COVID-19 infection and compliance with mitigation measures, this relationship was explored across the respondents’ gender, age group, income quantiles and levels of educational attainment. A country-level analysis was also considered in this section, given the potential variation in the association between concern about COVID-19 infection and compliance with safety practices, due to differences in the policies implemented in the various countries.

#### 3.2.1. Gender

**Fig 1:**
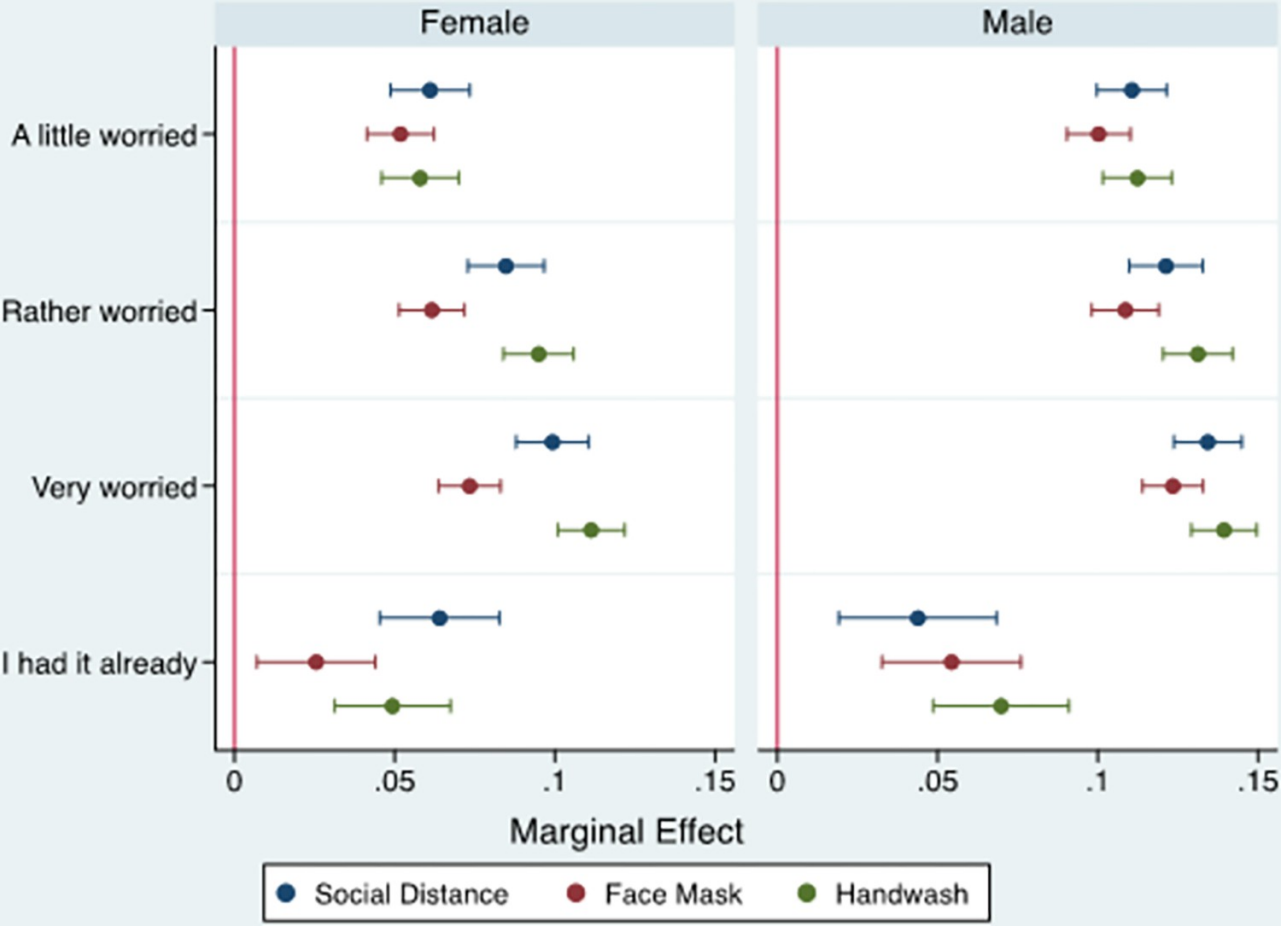
Marginal effect of individuals’ worriedness about COVID-19 infection on compliance with mitigation measures by gender.

Fig 1 (and S2 Table) show the marginal effects of concern about infection on the probabilities of adhering to COVID-19 mitigation measures by gender. Both the sign and magnitude of coefficients related to the association between the level of an individual’s concern and adherence to repeated handwashing and maintaining of social distancing were qualitatively similar to those reported by the pooled model (Table 2). However, the results notably showed that the marginal effect of concern about infection on the use of face masks was higher for men than women across all categories of the independent variable, implying that men were more likely to adhere to these particular proactive behaviours.

#### 3.2.2. Age group

**Fig 2:**
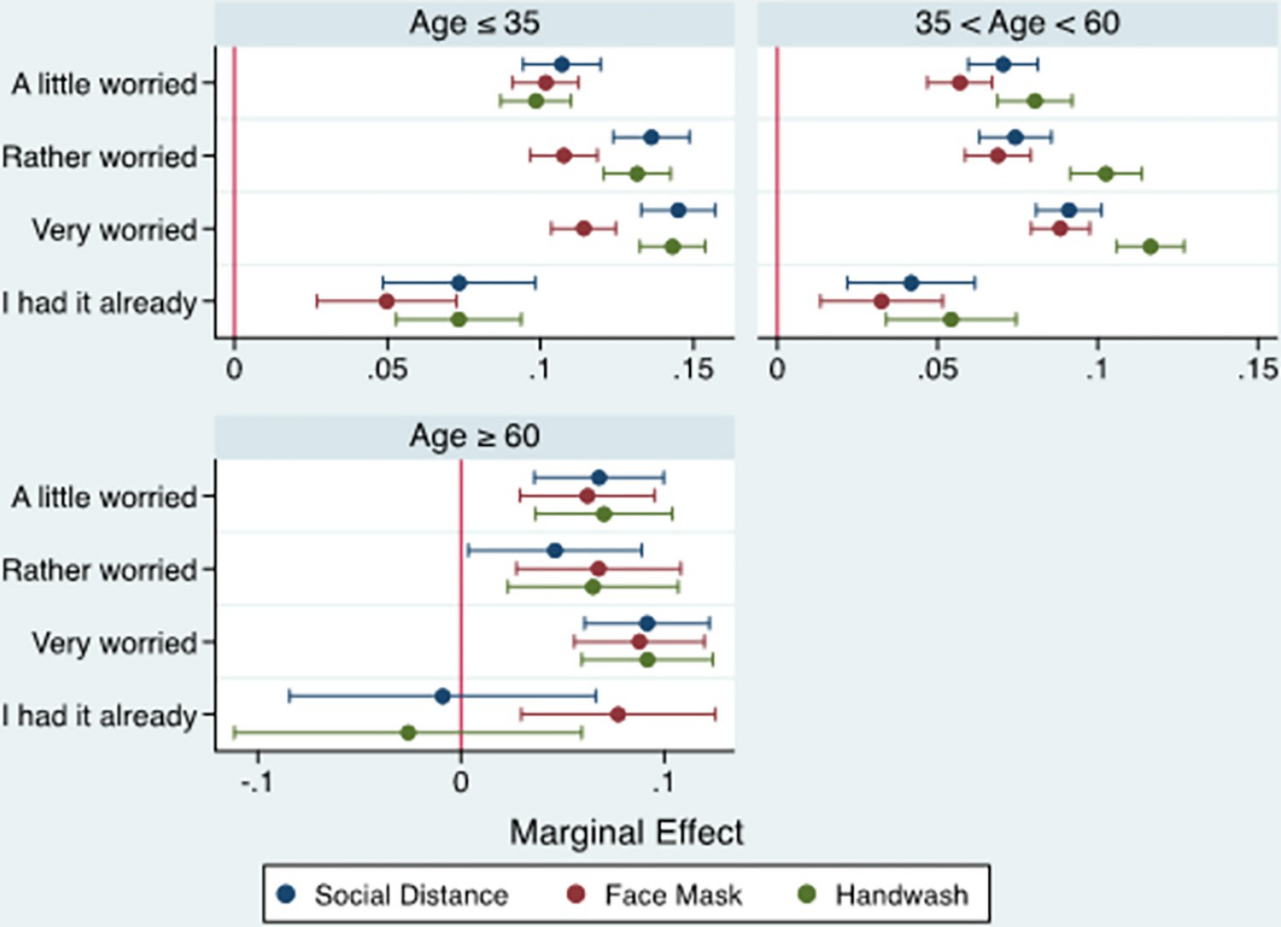
Marginal effect of individuals’ worriedness about COVID-19 infection on compliance with mitigation measures by age.

Fig 2 (and S3 Table) present the marginal effects of the probit model by age group. Consistent with the full-sample estimates, the results confirmed that individuals who felt a high level of concern about infection (*very worried*) across all age groups were more likely to comply with Covid-19 mitigation measures, compared with those who perceived little or moderate levels of concern. A comparison of the magnitudes of the coefficients related to the three mitigation measures across age groups revealed that individuals belonging to the age group 35 years and below were more likely to follow all the mitigation measures, compared with other individuals in the age groups over 35 years. Interestingly, the results showed that younger individuals (under 35) were more compliant with recommendations for social distancing and using face masks than the elderly (over 60).

#### 3.2.3. Educational attainment

**Fig 3:**
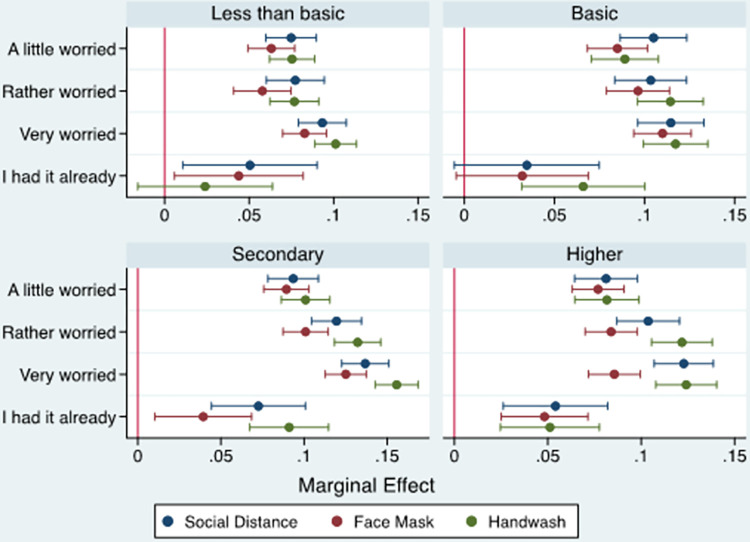
Marginal effect of individuals’ worriedness about COVID-19 infection on compliance with mitigation measures by education level.

Fig 3 (and S4A and S4B Table) display the marginal effects of concern about COVID-19 infection on the probabilities of compliance with COVID-19 mitigation measures across levels of education. The results revealed that the association between concern about infection and adherence to COVID-19 mitigation measures was highly significant in that the marginal effect in relation to the three preventive measures rose, with a few exceptions, as the level of education increased from less than basic and basic education to secondary and higher education levels.

#### 3.2.4. Income quantile

**Fig 4:**
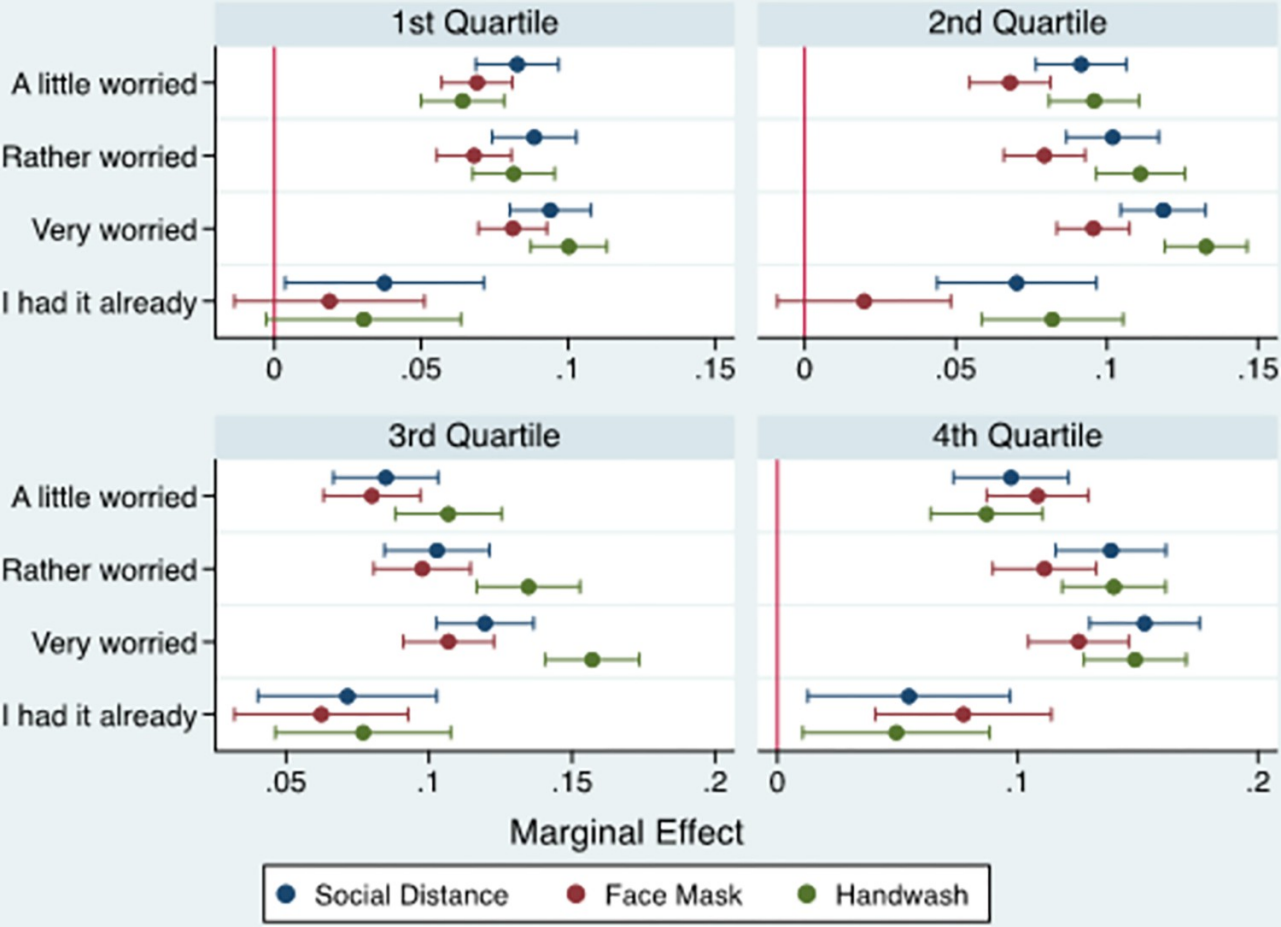
Marginal effect of individuals’ worriedness about COVID-19 infection on compliance with mitigation measures by income quantile.

Fig 4 (and S5A and S5B Table) report the estimated marginal effect of concern about COVID-19 infection on individuals’ compliance with mitigation measures across income groups. The results showed that individuals in the third and fourth-income quantiles were more likely to comply with the three mitigation measures than those in the lower income quantiles, providing a strong case for the effect of household income on individuals’ compliance with COVID-19 mitigation measures. In particular, the marginal effects of compliance with the mitigation measures in relation to the use of face masks were highest in absolute terms, indicating that the higher a household income was, the greater the probability of an individual following government recommendations regarding the wearing of face masks in public places during the pandemic.

#### 3.2.5. Country-level analysis

Tables 3 and 4 summarise the estimated marginal effect of concern about COVID-19 infection on individuals’ compliance with mitigation measures by country. The results showed distinct differences between the surveyed countries in relation to the influence of concern about infection on public compliance with the implemented preventive measures, with the strongest association being observed in Tunisia and Sudan and the lowest in Jordan and Morocco. A noteworthy observation from Tables 3 and 4 is that except for Morocco, the results confirmed the findings presented in Table 2 regarding the “first-up-then-down” trend of the odds of compliance with COVID-19 mitigation measures, which increased with individuals’ level of concern about infection from “a little worried” to “very worried”, and then fell when individuals were infected by the virus.

**Table 3:** Marginal effect of individuals’ concern about COVID-19 infection on compliance with mitigation measures by country.

Concern about infection^♣^	Jordan			Morocco			Sudan		
	Social distancing	Face mask	Hand washing	Social distancing	Face mask	Hand washing	Social distancing	Face mask	Hand washing
**A little worried**	0.071***	0.044***	0.094***	0.077***	0.061***	0.059***	0.100***	0.144***	0.090***
	(0.007)	(0.006)	(0.010)	(0.007)	(0.005)	(0.006)	(0.020)	(0.018)	(0.016)
**Rather worried**	0.096***	0.057***	0.138***	0.051***	0.044***	0.044***	0.159***	0.126***	0.150***
	(0.007)	(0.005)	(0.009)	(0.011)	(0.008)	(0.009)	(0.019)	(0.018)	(0.014)
**Very worried**	0.102***	0.068***	0.153***	0.069***	0.048***	0.060***	0.222***	0.168***	0.141***
	(0.007)	(0.005)	(0.009)	(0.007)	(0.006)	(0.006)	(0.017)	(0.017)	(0.013)
**Already infected**	0.064***	0.028***	0.066***	0.034*	0.040***	0.052***	-0.212***	-0.074	-0.086
	(0.009)	(0.007)	(0.013)	(0.018)	(0.012)	(0.012)	(0.072)	(0.067)	(0.065)
**Observations**	7,471	7,410	7,471	8,120	8,120	8,120	4,401	4,401	4,401
**Controls**	YES	YES	YES	YES	YES	YES	YES	YES	YES
**Country &Admin FE**	YES	YES	YES	YES	YES	YES	YES	YES	YES
**Wave FE**	YES	YES	YES	YES	YES	YES	YES	YES	YES
**Pseudo R** ^**2**^	0.0780	0.108	0.0624	0.0805	0.121	0.107	0.0574	0.0903	0.0602
**Wald chi** ^**2**^	375.5	313.7	426.2	427	437.9	467.1	334	489.1	270.4

^♣^ Reference group is “not worried”. Standard errors in parentheses

*** p<0.01

** p<0.05

* p<0.1. Household size, urban, gender, age, marital status, employment status, education, wave, country and administrative fixed effects were controlled for in all the models.

**Table 4:** Marginal effect of individuals’ concern about COVID-19 infection on compliance with mitigation measures by country.

Concern about infection^♣^	Tunisia			Egypt		
	Social distancing	Face mask	Hand washing	Social distancing	Face mask	Hand washing
**A little worried**	0.091***	0.095***	0.108***	0.092***	0.070***	0.090***
	(0.007)	(0.007)	(0.008)	(0.011)	(0.010)	(0.011)
**Rather worried**	0.109***	0.120***	0.141***	0.105***	0.090***	0.103***
	(0.007)	(0.007)	(0.007)	(0.010)	(0.010)	(0.010)
**Very worried**	0.121***	0.139***	0.153***	0.116***	0.105***	0.148***
	(0.006)	(0.006)	(0.007)	(0.010)	(0.009)	(0.009)
**Already infected**	0.039***	0.059***	0.067***	0.091***	0.053***	0.101***
	(0.015)	(0.015)	(0.015)	(0.016)	(0.018)	(0.015)
**Observations**	7,437	7,437	7,437	4,000	4,004	4,003
**Controls**	YES	YES	YES	YES	YES	YES
**Country / Admin FE**	YES	YES	YES	YES	YES	YES
**Wave FE**	YES	YES	YES	YES	YES	YES
**Pseudo R** ^**2**^	0.152	0.177	0.113	0.0812	0.0860	0.0873
**Wald chi** ^**2**^	693.1	845.4	621.6	242.9	243.9	263

^♣^ Reference group is “not worried”. Standard errors in parentheses

*** p<0.01

** p<0.05

* p<0.1. Household size, urban, gender, age, marital status, employment status, education, wave, country and administrative fixed effects were controlled for in all the models

## 4. Discussion

Overall, the empirical results provided plausible evidence of the impact of risk perception on individuals’ risk-related behaviour. The probit model estimates revealed a positive and statistically significant association between level of concern about contracting COVID-19 and individual compliance with the recommended measures implemented by the governments of the surveyed countries for risk prevention and mitigation. This is consistent with the findings of previous studies in this field, which suggest that perceived risks and fears of infection are dominant predictors of compliance with public health measures [e.g. 24, 31]. In other words, individuals with higher levels of fear and risk perception are often more concerned, anxious and stressed, and therefore more likely to follow and comply with preventive measures such as handwashing, using face masks and social distancing.

Interestingly, adherence to COVID-19 mitigation measures were found to follow a “first-up-then-down” trend, while compliance with the three mitigation measures went hand in hand with the level of individuals’ concern about contracting COVID-19, but then markedly decreased after individuals contracted the virus. This finding supports those of Lio et al. [32] and Binte et al. [33], who revealed that individuals with a positive COVID-19 history were less likely to adhere to mitigation measures compared with uninfected individuals who have no prior history of infection. This tends to imply that individuals who were infected and then recovered perceived the disease as posing less risk, which subsequently reduced their protective behaviour [34]. Another explanation is that individuals who were infected may have had lower levels of compliance because of the sense of security that recovery from infection may provide [35]. However, research on previous disease outbreaks indicates that sharp decreases in risk perception can contribute to reinfections and the emergence of much severer subsequent waves of infection that might cause high mortality rates and have serious economic effects [36]. For instance, previous research has shown that lower levels of compliance with risk mitigation measures together with relaxed or no lockdown measures contributed to an increase in the rates of reinfection in several countries. These were estimated by Ribeiro Xavier et al. [37] to be as high as 40% in South Africa, and by Hoang [38] to be 14% in China and 31% in Korea.

In line with the recent literature on COVID-19 risk perception and adoption of preventive measures [24, 39], the results of the present study showed that individual attributes (age, gender and education) and household size were key predictors of individuals’ compliance with public health measures. The finding that females were significantly more likely than their male counterparts to adhere to the three outcome measures is consistent with that of Ning et al. [40], who point out that women generally tend to have a higher level of concern and fear of infection, and a greater sense of responsibility and willingness to protect society, making them keener and more likely to engage in recommended health behaviours than men. Likewise, the results revealed that older adults were more likely than younger adults to adopt protective behaviours. This supports the findings of Bronfman et al. [41], who illustrate that older adults perceive themselves to be more vulnerable to disease infections, especially in the presence of comorbidities, which motivates them to embrace protective behaviours, compared with younger adults, to avoid worse outcomes of COVID-19 infections.

In relation to the role of educational attainment, individuals with a basic level of education were found to be significantly less likely to comply with mitigation measures than others with a less than basic level of education. In this respect, Lieberoth et al. [42] report similar findings, while their results revealed that people with lower education levels tended to feel more anxious and stressed by the COVID-19 pandemic, and thus were more likely to follow the recommended preventive measures. However, the present study found evidence that individuals with higher educational degrees were significantly more likely than those with less than basic levels of education to adhere to the recommended mitigation measures during the COVID-19 pandemic. This finding is in agreement with Oyetunji et al. [43], who point out that having more than twelve years of formal schooling is positively associated with practising more than one protective measure to prevent infection.

In general, the results related to household size in the three models indicated that a larger household size hampers individuals’ ability to comply with mitigation measures. In this respect, Ye et al. [44] investigated individual and household factors influencing the adoption of preventive behaviours among a large sample of households from eight provinces in China, and found that members of larger households had limited capacities to adopt COVID-19 preventive measures. In particular, the statistically highly significant coefficient of this variable in the second model (using a face mask), in contrast to the handwashing and social distancing models, can be attributed to the nature of this behaviour in the sense that several MENA countries experienced shortages of medical masks during the early stages of the pandemic [45]. It is also the only preventive behaviour among the three investigated behaviours that involves a direct financial cost [46], which may be unaffordable for larger households. Finally, no statistically significant evidence was found of the impact of the characteristics of the community (rural vs. urban areas) in which an individual resides on compliance with COVID-19 preventive and mitigation measures.

Turning to the results of the heterogeneity analysis, both the sign and magnitude of coefficients in the estimated gender-based models were qualitatively similar to those reported by the pooled model, and conformed with previous studies showing that women are more likely than men to follow proactive health measures during public health crises [41]. One possible explanation for this is that males’ usual social and professional or labour activities lead them to leave home more often and socialise more frequently, which had implications for their adoption of preventive behaviours [40]. However, the results showed that the association between concern about infection and use of face masks was stronger among men than women across all categories of the independent variable. A study by Balkhi et al. [47] in the context of Pakistan ascribed similar findings to certain cultural and environmental factors as men are most often the breadwinners in the household, and thus had to comply with mandatory measures relating to maintaining social distancing and wearing face masks in workplaces and public places. In the same vein, Alghalyini et al. [48] demonstrate that women in Saudi Arabia, a country in the MENA region, were less likely than men to leave their home during the pandemic and conducted more work-related and household tasks from home (e.g. working from home and online shopping). This minimised their interactions with others in public places and reduced the likelihood that they would need to comply with mandatory measures of using face masks in public spaces and workplaces.

The age-differentiated estimates confirmed the full-sample results that higher levels of concern about infection were associated with greater likelihoods of complying with Covid-19 mitigation measures across all age groups. Notably, the finding that younger individuals (under 35) were more likely to follow all the mitigation measures than individuals over 35 slightly contradicts previous studies, which show that middle-aged individuals reported higher levels of compliance with COVID-19 preventive behaviours than their younger counterparts [18]. However, the results showed that the elderly (over 60) were less compliant with social distancing and face mask recommendations than younger individuals (under 35). This finding is in agreement with the findings of Alahdal et al. [49], showing a significant difference in the level of compliance with COVID-19 proactive measures with better practices observed among younger individuals (18–49 years) compared with those aged over 60. This was attributed to their greater use of various social media channels compared with the elderly.

In terms of the influence of educational attainment on the association between concern about infection and adherence to COVID-19 mitigation measures, it was found that, with a few exceptions, the likelihood of an individual’s compliance with the three preventive measures increased with the level of the individual’s educational attainment. This finding is in agreement with previous studies indicating that lower education levels are barriers to compliance with COVID-19 preventive measures [50], and that individuals with higher education levels are more aware of and more anxious about the COVID-19 pandemic, which increased the probability of their adoption of COVID-19 preventive measures and precautionary practices [51].

A comparison across income groups revealed that individuals belonging to higher income quantiles (i.e. the third- and fourth-income quantiles) were more likely to comply with the three mitigation measures than those in the lower income quantiles. In particular, this was the case for the use of face masks, where income level was a highly significant predictor of individuals’ adherence to government recommendations regarding the wearing of face masks in public places during the pandemic. This finding is in agreement with the wide array of studies in this field that have found evidence of the role of income in predicting individuals’ adherence to COVID-19 proactive and mitigation measures [18, 52]. For instance, El-Shal and Moustafa [53] show that economic hardship moved people in MENA away from adhering to COVID-19 mitigation measures as they became less concerned about being infected with COVID-19, perceiving that they had little to lose given their already precarious existence. Alkhaldi et al. [54] point out that low-income households often encounter significant barriers in relation to the adoption of preventive measures because of their crowded living situation, involvement in the informal labour market, and inability to afford the cost of hygiene products, which collectively makes them less able and willing to comply with these mitigation measures. Shahin and Hussein [52] illustrate that the purchasing of mitigation and precautionary equipment during the COVID-19 pandemic (e.g. alcohol, detergents and face masks) represented an additional monetary burden for many Egyptian households, whose income and financial status had already worsened due to the adverse impacts of the pandemic on employment, incomes and labour markets.

Finally, the results revealed distinct differences across the surveyed countries regarding the association between the level of concern about contracting COVID-19 infection and public compliance with the preventive and mitigation measures. While many previous studies on MENA countries have often regarded the region as a single homogeneous unit [e.g. 14, 15], this finding underscores the issues that may result from ignoring the fact that MENA is a highly diverse region, incorporating low, middle and high-income countries at different stages of socio-demographic transition. It also contains socio-politically stable countries and others with ongoing conflicts and emergencies. Neglecting these differences precludes the possibility of analysing the relationship between concern and compliance from a deeper perspective and makes a generalisation of the results questionable. In this regard, existing evidence attributes heterogeneities in compliance with COVID-19 mitigation measures between countries to a suite of governance and institutional factors (e.g. the stringency of the measures, their length of implementation and the degree of social trust in governments), and a range of socioeconomic factors (e.g. the demographic composition of the population, including age and gender, social capital, the degree of ethnic diversity, income level), and political beliefs, and other cultural beliefs and social characteristics among the population [55, 56]. Therefore, heterogeneities among MENA countries in relation to the varying levels of the infrastructures of health systems have arguably created specific challenges for individual countries and influenced the provisions of public health policies during the pandemic, led to uneven responses by governments in terms of the implementation of risk management measures, and largely determined people’s compliance with the enforced containment measures [57].

Despite the consistency and relevance of the present study’s results, three limitations of the present study should be noted and considered in future research. First, the major limitation is that the CCMMHH survey was designed for other purposes and without reference to the empirical model, which indeed limited the efficacy of this model. For instance, psychological models of behaviour change, including the HBM that formed the theoretical underpinnings of this study, suggest that individual adoption of protective behaviours rests on a much wider array of factors than just risk perception. Owing to the limitations of the survey data, some important predictors of behaviour were excluded. Future research would benefit from extending these results and fleshing out the model by accounting for psychological predictors (e.g. risk attitudes, beliefs, self-efficacy and perceived behavioural control) and contextual variables (e.g. economic, socio-political and temporal factors) that may influence public ability and willingness to comply with risk preventive mitigation measures. A second limitation of this study is that the sample was obtained through a telephone panel survey, which subjects the results to the biases that this context might entail. Finally, the third limitation is related to potential endogeneity of the concern about infection variable, which prevents a claim of a clausal relationship.

## 5. Conclusions and policy implications

Using a panel dataset based on five waves of the Combined COVID-19 MENA Monitor Household Survey (CCMMHH) conducted in Jordan, Morocco, Sudan, Tunisia and Egypt between November 2020 and August 2021, this study examined the association between individuals’ worries about contracting COVID-19 and their adherence to the recommended preventive measures of wearing a face mask, maintaining social distancing and handwashing. The empirical findings provide evidence about community practices around the use of risk prevention and mitigation measures during public health crises in settings with different infection incidences and response policies, which should inform policies that aim to mitigate the transmission of infectious diseases and combat their negative externalities. Specifically, a number of conclusions and policy implications can be drawn for effective risk communication and management during disease outbreaks and public health emergencies to encourage appropriate public health behaviours.

First, the overall results provide plausible evidence of the impact of risk perception on individuals’ risk-related behaviours, with the probit model estimates revealing a positive and statistically significant association between level of concern about contracting COVID-19 and individual compliance with the recommended measures implemented by the governments of the surveyed countries for risk prevention and mitigation. Government interventions aimed at promoting the adoption of risk mitigation measures during pandemics and disease outbreaks should tailor messages more effectively to communicate health risks to susceptible segments of the population and enhance their perceptions of threat and self-efficacy, and thereby encourage them to perform certain behaviours. In the same vein, government campaigns should also focus on boosting public attitudes and knowledge by nudging them toward trusted sources of information about causes of infection and associated health risks to prevent negative outcomes of conspiracy theorising in times of public health crises. To this end, building trust in government authorities is essential in order to influence the perception of risk and promote the adoption of preventive public health measures.

Second, the results highlight a decreasing trend in the association between concern about COVID-19 infection and compliance with preventive and mitigation measures after an individual has contracted the virus. This implies that worries about infection decreased as the pandemic continued, and that when people became more familiar with the pandemic, including by contracting the virus, they perceived fewer health risks and thus adopted less strict hygienic behaviours. This should be a cause of concern with regard to the effectiveness of social protection measures that the governments of the surveyed countries implemented to mitigate the economic impacts of their mitigation measures, which seem to have created a dilemma between complying with the preventive measures and maintaining a source of income. In connection with this, a look at public policy discussions in relation to the COVID-19 pandemic in MENA countries, especially at the beginning of the outbreak, shows that they were dominated by health concerns, whereas little focus was given to the economic side-effects of the implemented mitigation measures, which might be riskier than the virus. Such false trade-offs, which prioritised health and ignored the economy, seem to have reduced acceptance of the implemented measures among people with lower incomes and other marginalised groups in the population. Therefore, these findings imply that risk mitigation interventions and communication approaches need to shift from a context of emergency and uncertainty to one that encourages sustainable and habitual behaviours. Considerations within government interventions must address the wider impacts of disease control measures by including dimensions beyond biomedical risks that account for the social and economic effects of these measures on the population over time, as these effects may act as a deterrent to public compliance with risk prevention and mitigation measures.

Third, the results clearly point to the existence of remarkable variations between the individuals in the surveyed sample in relation to the determinants of their compliance with COVID-19 mitigation measures based on their age, gender and other socio-demographic and economic characteristics. A deeper understanding of the perceptions and attitudes towards mitigation behaviours and their underlying drivers among different socio-demographic groups is a key element in bringing about behavioural changes and promoting compliance with these measures. In conjunction with this, more research is needed to understand the communication preferences and trusted sources and channels of information in order to design tailored interventions for each target group. In this respect, it is essential to map the key trusted influencers for each group and engage them in crafting tailored communication and nudging messages to ensure that desired behavioural changes are effectively accomplished. Such strategies are more likely to be successful if they co-are designed through community-centred approaches that acknowledge local realities and meaningfully involve the target groups to identify and implement locally appropriate mitigation measures.

Fourth, the cross-country analysis reveals regional heterogeneities among the five surveyed countries in relation to the influence of COVID-19 concerns on public adherence to the implemented mitigation measures. This offers an opportunity for regional cooperation and collaboration between policymakers in public health sectors within the sampled countries. Specifically, policymakers in these countries can capitalise on these heterogeneities to develop cross-government approaches and create platforms for ongoing dialogue among stakeholders to promote a sharing of experience about effective approaches and best practices in order to reinforce public acceptance and people’s willingness to adhere to preventive and mitigation measures.

## Supporting information

S1 Table Number of respondents to the CCMMHH survey from each country across the survey waves. Source: CCMMHH survey [44].
(PDF)

S2 Table Marginal effect of individuals’ worriedness about COVID-19 infection on compliance with mitigation measures by gender. ^♣^ Reference group is “not worried”. Standard errors in parentheses *** p<0.01, ** p<0.05, * p<0.1. We controlled for household size, urban, gender, education, marital status, employment status, income quartile, wave, country and administrative fixed effect in all the models.
(PDF)

S3 Table Marginal effect of individuals’ worriedness about Covid-19 infection on compliance with mitigation measures by age. ^♣^ Reference group is “not worried”. Standard errors in parentheses *** p<0.01, ** p<0.05, * p<0.1. We controlled for household size, urban, gender, education, marital status, employment status, income quartile, wave, country and administrative fixed effect in all the models.
(PDF)

S4 Table (a and b). Marginal effect of individuals’ worriedness about COVID-19 infection on compliance with mitigation measures by education level. ^♣^ Reference group is “not worried”. Standard errors in parentheses *** p<0.01, ** p<0.05, * p<0.1. We controlled for household size, urban, gender, education, marital status, employment status, income quartile, wave, country and administrative fixed effect in all the models.
(PDF)

S5 Table (a and b). Marginal effect of individuals’ worriedness about Covid-19 infection on compliance with mitigation measures by income quantile. ^♣^ Reference group is “not worried”. Standard errors in parentheses *** p<0.01, ** p<0.05, * p<0.1. We controlled for household size, urban, gender, education, marital status, employment status, income quartile, wave, country and administrative fixed effect in all the models.
(PDF)
