# Supplementary material for: COVID-19 risk perception and public compliance with preventive measures: Evidence from a multi-wave household survey in the MENA region
Source: PLoS One. 2023 Jul 10;18(7):e0283412. doi: 10.1371/journal.pone.0283412 (PMC10332611; doi:10.1371/journal.pone.0283412)
Supplement: S1 Table — Source: CCMMHH survey [44]. (PDF) [file pone.0283412.s001.pdf]

S1 Table. Number of respondents to the CCMMHH survey from each country across the survey waves

Country	Survey waves				
	Wave I	Wave II	Wave III	Wave IV	Wave IIV
**Egypt**	1,923	2,000		2,007	
**Jordan**		2,549		2,503	2,573
**Morocco**	2,007	2,002	2,105	2,006	
**Tunisia**	2,000	2,077	2,057	2,009	
**Sudan**			2,400		2,001

Source: CCMMHH survey [44].
