# Supplementary material for: COVID-19 risk perception and public compliance with preventive measures: Evidence from a multi-wave household survey in the MENA region
Source: PLoS One. 2023 Jul 10;18(7):e0283412. doi: 10.1371/journal.pone.0283412 (PMC10332611; doi:10.1371/journal.pone.0283412)
Supplement: S2 Table — ♣ Reference group is “not worried”. Standard errors in parentheses *** p<0.01, ** p<0.05, * p<0.1. We controlled for household size, urban, gender, education, marital status, employment status, income quartile, wave, country and administrative fixed effect in all the models. (PDF) [file pone.0283412.s002.pdf]

S2 Table. Marginal effect of individuals' worriedness about COVID-19 infection on compliance with mitigation measures by gender

Worriedness about infection♣	Females			Males		
	Social Distance	Face Mask	Hand Wash	Social Distance	Face Mask	Hand Wash
**A little worried**	0.111***	0.052***	0.112***	0.061***	0.100***	0.058***
	(0.006)	(0.005)	(0.005)	(0.006)	(0.005)	(0.006)
**Rather worried**	0.121***	0.062***	0.131***	0.085***	0.109***	0.095***
	(0.006)	(0.005)	(0.006)	(0.006)	(0.005)	(0.006)
**Very worried**	0.134***	0.073***	0.139***	0.099***	0.123***	0.111***
	(0.005)	(0.005)	(0.005)	(0.006)	(0.005)	(0.005)
**Already infected**	0.044***	0.025***	0.070***	0.064***	0.054***	0.049***
	(0.013)	(0.009)	(0.011)	(0.010)	(0.011)	(0.009)
**Observations**	12,985	12,877	12,820	18,373	18,410	18,410
**Controls**	YES	YES	YES	YES	YES	YES
**Country & Admin FE**	YES	YES	YES	YES	YES	YES
**Wave FE**	YES	YES	YES	YES	YES	YES
**Pseudo R2**	0.157	0.187	0.0978	0.144	0.186	0.0902
**Wald chi2**	1521	1421	788.9	2235	2556	1427

♣ Reference group is "not worried". Standard errors in parentheses *** p<0.01, ** p<0.05, * p<0.1. We controlled for household size, urban, gender, education, marital status, employment status, income quartile, wave, country and administrative fixed effect in all the models
