# Supplementary material for: COVID-19 risk perception and public compliance with preventive measures: Evidence from a multi-wave household survey in the MENA region
Source: PLoS One. 2023 Jul 10;18(7):e0283412. doi: 10.1371/journal.pone.0283412 (PMC10332611; doi:10.1371/journal.pone.0283412)
Supplement: S3 Table — ♣ Reference group is “not worried”. Standard errors in parentheses *** p<0.01, ** p<0.05, * p<0.1. We controlled for household size, urban, gender, education, marital status, employment status, income quartile, wave, country and administrative fixed effect in all the models. (PDF) [file pone.0283412.s003.pdf]

S3 Table. Marginal effect of individuals' worriedness about Covid-19 infection on compliance with mitigation measures by age

	Age ≤ 35			35 < Age < 60			Age ≥ 60		
VARIABLES	Social Distance	Face Mask	Hand Wash	Social Distance	Face Mask	Hand Wash	Social Distance	Face Mask	Hand Wash
***Worried about infection*♣**									
A little worried	0.107***	0.102***	0.099***	0.070***	0.057***	0.080***	0.068***	0.062***	0.070***
	(0.006)	(0.005)	(0.006)	(0.006)	(0.005)	(0.006)	(0.016)	(0.017)	(0.017)
Rather worried	0.136***	0.108***	0.132***	0.074***	0.069***	0.102***	0.046**	0.068***	0.065***
	(0.006)	(0.006)	(0.006)	(0.006)	(0.005)	(0.006)	(0.022)	(0.021)	(0.021)
Very worried	0.145***	0.114***	0.143***	0.091***	0.088***	0.116***	0.091***	0.088***	0.092***
	(0.006)	(0.005)	(0.005)	(0.005)	(0.005)	(0.005)	(0.016)	(0.016)	(0.016)
Already infected	0.073***	0.050***	0.073***	0.042***	0.032***	0.054***	-0.009	0.077***	-0.026
	(0.013)	(0.012)	(0.010)	(0.010)	(0.010)	(0.010)	(0.038)	(0.024)	(0.044)
Observations	16,382	16,413	16,410	13,409	13,443	13,442	1,285	1,136	1,254
Controls	YES	YES	YES	YES	YES	YES	YES	YES	YES
Country/Adm.FE	YES	YES	YES	YES	YES	YES	YES	YES	YES
Wave FE	YES	YES	YES	YES	YES	YES	YES	YES	YES
Pseudo R2	0.142	0.179	0.0976	0.125	0.182	0.0945	0.220	0.239	0.159
Wald chi2	2190	2220	1327	1140	1428	920.1	228	198.4	133.2

♣ Reference group is "not worried". Standard errors in parentheses *** p<0.01, ** p<0.05, * p<0.1. We controlled for household size, urban, gender, education, marital status, employment status, income quartile, wave, country and administrative fixed effect in all the models.
