# Supplementary material for: COVID-19 risk perception and public compliance with preventive measures: Evidence from a multi-wave household survey in the MENA region
Source: PLoS One. 2023 Jul 10;18(7):e0283412. doi: 10.1371/journal.pone.0283412 (PMC10332611; doi:10.1371/journal.pone.0283412)
Supplement: S4 Table — (a and b). Marginal effect of individuals’ worriedness about COVID-19 infection on compliance with mitigation measures by education level. ♣ Reference group is “not worried”. Standard errors in parentheses *** p<0.01, ** p<0.05, * p<0.1. We controlled for household size, urban, gender, education, marital status, employment status, income quartile, wave, country and administrative fixed effect in all the models. (PDF) [file pone.0283412.s004.pdf]

S4 Table (a). Marginal effect of individuals' worriedness about COVID-19 infection on compliance with mitigation measures by education level

Worriedness about infection♣	Less than basic education			Basic education		
	Social Distance	Face Mask	Hand Wash	Social Distance	Face Mask	Hand Wash
**A little worried**	0.075***	0.063***	0.075***	0.011***	0.085***	0.089***
	(0.008)	(0.007)	(0.007)	(0.009)	(0.009)	(0.010)
**Rather worried**	0.077***	0.058***	0.077***	0.103***	0.096***	0.114***
	(0.009)	(0.009)	(0.007)	(0.010)	(0.009)	(0.009)
**Very worried**	0.093***	0.083***	0.101***	0.115***	0.110***	0.117***
	(0.007)	(0.007)	(0.006)	(0.009)	(0.008)	(0.009)
**Already infected**	0.051**	0.044**	0.024	0.035*	0.032*	0.066***
	(0.020)	(0.019)	(0.020)	(0.020)	(0.019)	(0.017)
**Observations**	6,925	6,919	6,963	5,662	5,708	5,728
**Controls**	YES	YES	YES	YES	YES	YES
**Country /Admin FE**	YES	YES	YES	YES	YES	YES
**Wave FE**	YES	YES	YES	YES	YES	YES
**Pseudo R2**	0.171	0.241	0.157	0.143	0.217	0.0991
**Wald chi2**	805.9	993.4	644.2	626.4	804.4	425.8

♣ Reference group is "not worried". Standard errors in parentheses *** p<0.01, ** p<0.05, * p<0.1. We controlled for household size, urban, gender, age, marital status, employment status, income quartile, wave, country and administrative fixed effect in all the models

S4 Table (b). Marginal effect of individuals' worriedness about COVID-19 infection on compliance with mitigation measures by education level

Worriedness about infection♣	Secondary education			Higher education		
	Social Distance	Face Mask	Hand Wash	Social Distance	Face Mask	Hand Wash
**A little worried**	0.093***	0.089***	0.101***	0.081***	0.077***	0.082***
	(0.008)	(0.007)	(0.007)	(0.009)	(0.007)	(0.009)
**Rather worried**	0.119***	0.101***	0.132***	0.104***	0.084***	0.122***
	(0.008)	(0.007)	(0.007)	(0.009)	(0.007)	(0.008)
**Very worried**	0.137***	0.125***	0.156***	0.123***	0.086***	0.124***
	(0.007)	(0.006)	(0.007)	(0.008)	(0.007)	(0.008)
**Already infected**	0.073***	0.039***	0.091***	0.054***	0.048***	0.051***
	(0.014)	(0.015)	(0.012)	(0.014)	(0.012)	(0.014)
**Observations**	10,215	10,135	10,187	8,365	8,339	8,335
**Controls**	YES	YES	YES	YES	YES	YES
**Country /Admin FE**	YES	YES	YES	YES	YES	YES
**Wave FE**	YES	YES	YES	YES	YES	YES
**Pseudo R2**	0.154	0.174	0.0966	0.165	0.178	0.0809
**Wald chi2**	1372	1350	839.9	1138	1027	566.9

♣ Reference group is "not worried". Standard errors in parentheses *** p<0.01, ** p<0.05, * p<0.1. We controlled for household size, urban, gender, age, marital status, employment status, income quartile, wave, country and administrative fixed effect in all the models.
