# Supplementary material for: COVID-19 risk perception and public compliance with preventive measures: Evidence from a multi-wave household survey in the MENA region
Source: PLoS One. 2023 Jul 10;18(7):e0283412. doi: 10.1371/journal.pone.0283412 (PMC10332611; doi:10.1371/journal.pone.0283412)
Supplement: S5 Table — (a and b). Marginal effect of individuals’ worriedness about Covid-19 infection on compliance with mitigation measures by income quantile. ♣ Reference group is “not worried”. Standard errors in parentheses *** p<0.01, ** p<0.05, * p<0.1. We controlled for household size, urban, gender, education, marital status, employment status, income quartile, wave, country and administrative fixed effect in all the models. (PDF) [file pone.0283412.s005.pdf]

S5 Table (a). Marginal effect of individuals' worriedness about Covid-19 infection on compliance with mitigation measures by income quantile

Worriedness about infection♣	1st Quartile			2nd Quartile		
	Social Distance	Face Mask	Hand Wash	Social Distance	Face Mask	Hand Wash
**A little worried**	0.083***	0.069***	0.064***	0.091***	0.068***	0.096***
	(0.007)	(0.006)	(0.007)	(0.008)	(0.007)	(0.008)
**Rather worried**	0.088***	0.068***	0.081***	0.102***	0.079***	0.111***
	(0.007)	(0.006)	(0.007)	(0.008)	(0.007)	(0.008)
**Very worried**	0.094***	0.081***	0.100***	0.119***	0.095***	0.133***
	(0.007)	(0.006)	(0.007)	(0.007)	(0.006)	(0.007)
**Already infected**	0.038**	0.019	0.030*	0.070***	0.020	0.082***
	(0.017)	(0.016)	(0.017)	(0.013)	(0.015)	(0.012)
**Observations**	7,908	7,920	7,921	7,936	7,956	7,975
**Controls**	YES	YES	YES	YES	YES	YES
**Country / Admin FE**	YES	YES	YES	YES	YES	YES
**Wave FE**	YES	YES	YES	YES	YES	YES
**Pseudo R2**	0.125	0.189	0.109	0.142	0.179	0.104
**Wald chi2**	659.5	853.6	562.2	826.8	859.9	664.7

♣ Reference group is "not worried". Standard errors in parentheses *** p<0.01, ** p<0.05, * p<0.1. We controlled for household size, urban, gender, age, marital status, employment status, education, wave, country and administrative fixed effect in all the models.

S5 Table (b). Marginal effect of individuals' worriedness about Covid-19 infection on compliance with mitigation measures by income quantile

Worriedness about Covid-19 infection♣	3rd Quartile			4th Quartile		
	Social Distance	Face Mask	Hand Wash	Social Distance	Face Mask	Hand Wash
**A little worried**	0.085***	0.080***	0.107***	0.097***	0.108***	0.087***
	(0.009)	(0.009)	(0.009)	(0.012)	(0.011)	(0.012)
**Rather worried**	0.103***	0.098***	0.135***	0.139***	0.111***	0.140***
	(0.009)	(0.009)	(0.009)	(0.012)	(0.011)	(0.011)
**Very worried**	0.120***	0.107***	0.157***	0.153***	0.125***	0.149***
	(0.009)	(0.008)	(0.008)	(0.012)	(0.011)	(0.011)
**Already infected**	0.071***	0.062***	0.077***	0.055**	0.077***	0.049**
	(0.016)	(0.015)	(0.016)	(0.021)	(0.019)	(0.020)
**Observations**	6,191	6,119	6,202	5,391	5,344	5,410
**Controls**	YES	YES	YES	YES	YES	YES
**Country / Admin FE**	YES	YES	YES	YES	YES	YES
**Wave FE**	YES	YES	YES	YES	YES	YES
**Pseudo R2**	0.139	0.168	0.0940	0.178	0.173	0.0762
**Wald chi2**	710.3	766.1	512	954.3	814.9	415.3

♣ Reference group is "not worried". Standard errors in parentheses *** p<0.01, ** p<0.05, * p<0.1. We controlled for household size, urban, gender, age, marital status, employment status, education, wave, country and administrative fixed effect in all the models.
